# Predictors of the Level of Physical Activity in Physically Active Older People

**DOI:** 10.3390/bs12090331

**Published:** 2022-09-14

**Authors:** María Antonia Parra-Rizo, Jaime Vásquez-Gómez, Cristian Álvarez, Ximena Diaz-Martínez, Claudia Troncoso, Ana Maria Leiva-Ordoñez, Rafael Zapata-Lamana, Igor Cigarroa

**Affiliations:** 1 Faculty of Health Sciences, Valencian International University (VIU), 46002 Valencia, Spain; 2 Department of Health Psychology, Faculty of Social and Health Sciences, Campus of Elche, Miguel Hernandez University (UMH), 03202 Elche, Spain; 3 Centro de Investigación de Estudios Avanzados del Maule (CIEAM), Laboratorio de Rendimiento Humano, Universidad Católica del Maule, Talca 3460000, Chile; 4 Exercise and Rehabilitation Sciences Institute, School of Physical Therapy, Faculty of Rehabilitation Sciences, Universidad Andres Bello, Santiago 7591538, Chile; 5 Grupo de Investigación en Calidad de Vida, Departamento de Ciencias de la Educación, Facultad de Educación y Humanidades, Universidad del Biobío, Chillán 378000, Chile; 6 Centro de Investigación en Educación y Desarrollo (CIEDE-UCSC), Departamento de Salud Pública, Facultad de Medicina, Universidad Católica de la Santísima Concepción, Concepción 4090541, Chile; 7 Instituto de Anatomía, Histología y Patología, Facultad de Medicina, Universidad Austral de Chile, Valdivia 5090000, Chile; 8 Escuela de Educación, Universidad de Concepción, Los Ángeles 4440000, Chile; 9 Escuela de Kinesiología, Facultad de Salud, Universidad Santo Tomás, Los Ángeles 4440000, Chile

**Keywords:** elderly, physical activity, functional capacity, quality of life, leisure, gender, education level

## Abstract

There has not been much study of risk profiles in older people according to different levels of practice in physical activity. For this reason, the aim of this research was to evaluate whether the elements that influence the quality of life and factors such as gender and education can predict the level of physical activity in the physically active elderly population. The Fernández–Ballesteros quality of life questionnaire and the WHO International Physical Activity Questionnaire were applied to a sample of 397 people with a mean age of 69.65 years (*SD* = 4.71). The results revealed the following predictive factors of practicing a low level of physical activity (*p* < 0.05): being a woman; having a low educational level; and low scores in activity and leisure and in functional skills. In conclusion, gender, education, functional skills, activity and leisure, and health are elements of quality of life that predict the level of physical activity performed by the elderly, where it is necessary to use leisure activities (visiting friends, playing games, running errands) as an indirect way to increase participation in physical activity.

## 1. Introduction

The aging process causes physiological changes that can affect not only vulnerability, but also the development of physical and mental diseases. Good physical fitness is associated with a better quality of life, wellbeing, and mental health in the elderly [1]. Some authors [2] warn that the gradual aging of society also represents a burden for the public health of states.

Exercise training and physical activity are strategies that prevent many chronic diseases (stroke, diabetes, osteoporosis, obesity, among others). Both strategies improve mobility, mental health, and quality of life; reduce mortality [3]; and increase muscle strength, mass, and function [4]. They also represent non-pharmacological therapies for preventing cardiovascular diseases [5].

In this regard, some studies highlight that physical activity is one of the most important strategies for disease prevention and promotion of physical and cognitive health, and that the quality of life of the elderly depends, precisely, on aspects such as being active, enjoying good health, and having social relationships [6,7]. Other authors point out that performing regular physical activity, participating in leisure activities, sleeping between 7–8 h a day, and staying outside the BMI (body mass index) ranges considered as low weight or obesity are components that predict a healthy lifestyle and longer survival [8]. There is also research that adds factors such as age, educational level, income, and stress to physical activity [9].

In relation to better functional ability, the practice of physical activity was shown to be beneficial in 63 participants aged between 65 and 95 years [10], where functional ability, in turn, was one of the factors that influence adherence to the practice of physical activity [11,12]. In fact, older people with a high practice of physical activity obtained better levels of functional ability and autonomy, which also increased their independence to carry out basic activities of daily life, encouraged their social ties, and improved satisfaction with their health, with the consequent socio-emotional benefits [13]. By contrast, older adults with functional limitations were more likely to be physically inactive. Regarding physical activity, some researchers [14] indicate that its maximum expression between 65–75 years of age and decreases after 75 years of age.

When considering the variable gender, no studies have been found that address this matter, hence the novelty of the present work.

However, previous studies [15], albeit scarce, have paid attention to older women and the effectiveness of aerobic exercise on their health and quality of life. Thus, in a study carried out with 188 elderly people, it was found that women were more physically active than men, although men showed a higher quality of life [16]. Nor has the scientific literature dealt with gender differences linked to physical activity and falls that usually occur in older adults [17].

Likewise, and as previously mentioned, the practice of physical activity is related to social, cultural, and demographic aspects in older adults [18]. In this way, among the inequalities that influence physical activity levels, those related to educational level and income stand out. One study found that the prevalence of participation in physical activities of 9896 elderly people was higher in those with higher income and educational level, since they had a greater capacity to participate in activities associated with improving their health and welfare [19].

Similarly, in a study carried out with 689 people over 60 years of age, it was observed that people with a favourable financial position and education adopted significantly better health behaviours than the rest [20].

Therefore, the scientific literature mainly addresses health and functional ability as facilitating variables for the practice of physical activity in older people. However, this work also presents a predictive model on the causes of the low practice of physical activity based on variables that have been scarcely studied but are necessary, given their representativeness. In this regard, knowing the most descriptive variable of this model could allow us to adopt the necessary measures to encourage the practice of physical activity among the elderly, mitigating the inequalities that occur in this sector of the population.

Therefore, the purpose of this study is to analyse whether variables such as gender, educational level, and self-assessed quality of life can predict the level of physical activity performed by physically active older adults. The following research question has been posed: Does the level of physical activity undertaken by physically active older adults depend on their gender, the level of education they have attained, and the quality of life they enjoy?

## 2. Methods

### 2.1. Participants

In total, 397 elderly residents in Alicante (Spain) participated, with 64.7% women and 35.3% men. Participants were between 61 and 93 years old, and the mean age was 69.65 years (*SD* = 4.71). Regarding their marital status, 69.3% of the participants were married, 16.4% were widowed, 8.2% were single, and 6.1% were divorced. Regarding their living situation, 31.6% of the participants lived alone.

Considering the habits that can affect the health of the participants, regarding tobacco, 91.6% do not smoke, 1.8% smoke between 1 and 5 cigarettes a day, 4.3% smoke between 6 and 20 cigarettes a day, 1.5% smoke more than 20 cigarettes a day, and 0.8% only smoke on special occasions. Regarding alcohol consumption, 51.9% never drink it, 21.1% drink once a week, 20.2% drink daily, and 7.1% only drink on special occasions.

Regarding their state of health, 71.3% have no physical ailments and 87.9% have no psychological disorders. Among the modalities of sports practised by the participants, the following figures were found: 49.4% practice gymnastics, 24.4% practice yoga/pilates, 13.6% practice dance activities, and 12.6% practice aquatic activities. The inclusion criteria were (1) being over 60 years old, (2) being physically active, and (3) having been physically active for at least one year. The exclusion criterion was (1) having difficulties with reading and answering the questions in the questionnaires.

### 2.2. Instruments

#### 2.2.1. Sociodemographic Questionnaire

This study used a self-made instrument composed of 16 items related to gender, age, marital status, social and family environment, health status, type of coexistence, life habits of the participants, and types of sports practised.

#### 2.2.2. International Physical Activity Questionnaire, IPAQ, OMS, (2002)

This scale evaluates three types of physical activities: low-intensity activities, moderate-intensity activities, and vigorous-intensity activities. It classifies participants into three activity levels: high, moderate, and low. These different intensities of activities are considered as follows: <3 METs, 3–6 METs, and >6 METs, respectively [21]. Within the high activity-level people are those who do at least one more hour of moderate-intensity activity above their basal activity level per day, or half an hour of vigorous-intensity activity above their daily basal levels. Within the moderate activity level are those who do at least half an hour of moderate-intensity physical activity almost every day. Within the low activity level are those that are not in the moderate or high activity levels [22]. In the short version used in this research, the participant had to answer seven items related to the physical activity performed in the last seven days, with questions such as “Usually, how much time in total do you dedicate to intense physical activity on one of those days?” The reliability of the IPAQ short version is 0.65 (rs = 0.76; CI95%: 0.73–0.77). This questionnaire has been used in recent studies carried out on older people [23].

#### 2.2.3. Brief Quality of Life Questionnaire, CUBRECAVI [24]

For the evaluation of health, social integration, functional skills, and activity and leisure, the CUBRECAVI quality of life questionnaire was used. Based on the multidimensional concept of quality of life and health proposed by the WHO, this self-administered questionnaire evaluates the most relevant components of quality of life in older people. It is made up of 21 subscales, among which those used for this publication stand out. Participants must answer a Likert-type response scale regarding items such as ‘How do you think you can fend for yourself?’, where 0 is bad, 1 is regular, 2 is good, and 3 is very good. This questionnaire has a level of internal consistency on the scales (Cronbach’s alpha) that ranges between 0.70 and 0.92 [25]. It takes approximately 20 min to complete the questionnaire. It is a highly recommended questionnaire to assess the quality of life [25] and has recently been used in older people [26].

### 2.3. Procedure

The study was cross sectional. The selection of participants was carried out in two contexts in Alicante: in sports and social centres and in outdoor spaces where sport is practised regularly. In total, 38 centres were contacted, of which 18 agreed to collaborate. Those interested in participating were given a copy of the informed consent form and the questionnaire, which they completed individually after practising physical activity. In outdoor spaces where the population goes to practise sport, contact was made with people who met the requirements defined for the study, and the research and its objectives were explained to them. Those who agreed to participate were given an envelope containing the questionnaire and the informed consent form, which they had to return completed at a later appointment set at that time. Sampling was non-probabilistic by convenience.

This study was approved by the Ethics Committee of the Miguel Hernández University of Elche, Spain (200115191342; 2020.28.E.OIR), and the principles of the Helsinki Declaration of Ethics were followed.

### 2.4. Analysis of Data

A dependency analysis of the study variables was carried out. Student’s *t*-test for independent samples was applied to the continuous variables; a chi-squared test was applied to the categorical variables. To estimate the effect size, Cohen’s d (1988) was used in the *t*-tests for independent samples; in the qualitative variables, the Phi Coefficient and Cramer’s V (depending on the size of the contingency tables) were applied. 

Next, to study whether the variables analysed allow prediction of the level of physical activity of physically active elderly people, a logistic regression analysis was carried out using the method of entering. Then, the variables were dichotomized and the OR was calculated to assess the bivariate relationship of the predictor variables with physical activity.

The significance value established was <0.05.

The data analyses were performed with the SPSS statistical package, version 23.0. (IBM Corp., Armonk, NY, USA for Windows).

## 3. Results

The study sample was drawn from a population of physically active older people. It was found that 46.1% of the participants perform a high level of physical activity, 41.6% a moderate level, and 12.3% a low level. Based on these data, the physical activity level variable was dichotomized into two groups: 0 = high physical activity and 1 = low or moderate physical activity.

The association between the level of physical activity performed by the participants and the study variables has been analysed. On the one hand, the analyses collected in Table 1 show that the levels of physical activity practiced by the elderly are related to the scores they obtained in functional skills (*t* (383.656) = 4.819; *p* < 0.001; *d* = 0.46). and in activity and leisure (*t* (372.511) = 8.459; *p* < 0.001; *d* = 0.86). In addition, the participants’ health scores are not related to their activity level, although they are close to statistical significance (*t* (383.656) = 1.915; *p* = 0.056; *d* = 0.19).

The level of physical activity of the elderly is also related to gender (*χ*2(1, N = 397) = 9.293; *p* = 0.002; *Phi* = 0.153) and educational level (*χ*2(3, N = 397) = 33.140; *p* < 0.001; *V _Cramer_* = 0.289), but it is not associated with participants’ use of the socio-sanitary services (*χ*2(2, N = 397) = 0.676; *p* = 0.713; *V _Cramer_* = 0.041) (Table 2). 

**Figure 1:**
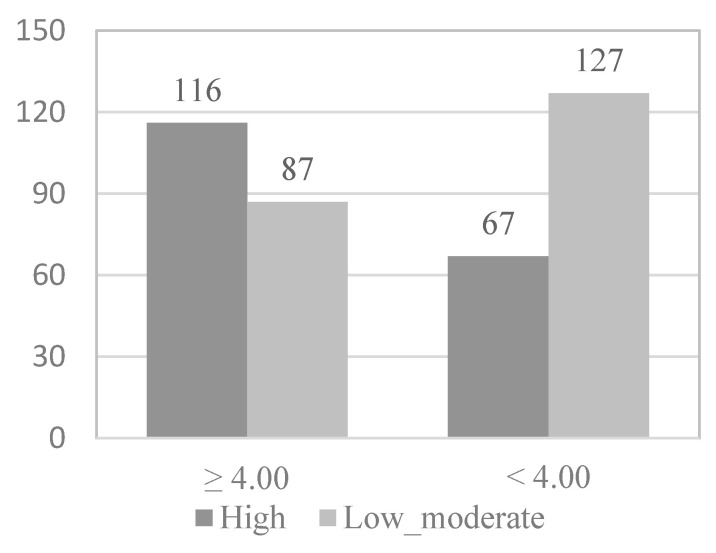
*Functional skills* physical activity level.

**Figure 2:**
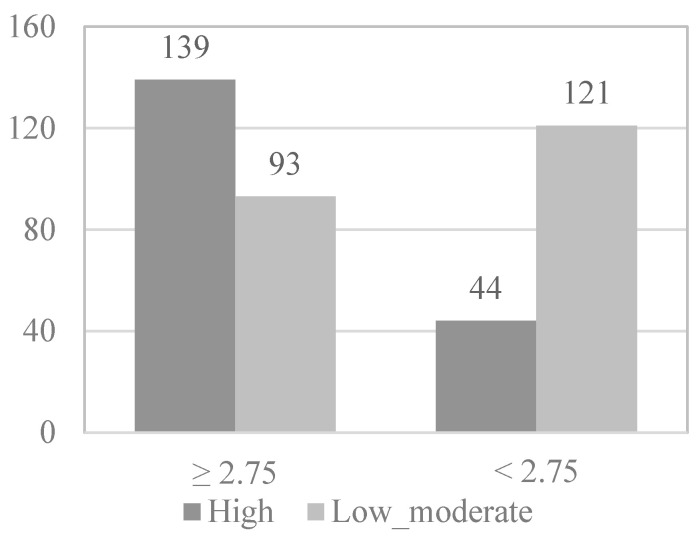
*Activity and leisure* physical activity level.

**Figure 3:**
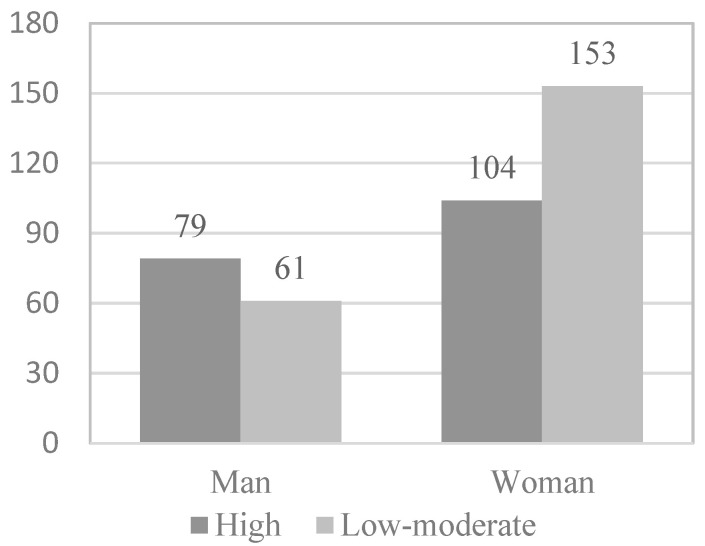
*Gender* physical activity level.

To study which of the variables that show or are close to showing statistical significance in the association analyses are predictive of the level of physical activity of the physically active elderly, the continuous variables were dichotomized by dividing the participants into two groups (0 and 1) according to the median. Following this principle, in the case of the functional skills scale (Figure 1), 203 elderly (51.1%) formed group 0, presenting high levels of functional skills (≥4.00), while 194 elderly (48.9%) formed group 1, presenting low levels of functional skills (<4.00). In the case of the activity and leisure scale (Figure 2), 232 elderly (58.4%) formed group 0, present high levels of activity and leisure (≥ 2.75), while 165 elderly (41.6%) formed group 1, presenting low levels of activity and leisure (<2.75). In the case of the health scale, 199 elderly (51.1%) formed group 0, presenting high levels of health (≥3.16), while 198 elderly (49.9%) formed group 1, presenting low levels of health (<3.16). In Figure 3, the information appears dichotomized by gender. Likewise, the education variable was dichotomized, grouping the participants who have received secondary or higher education in group 0 (38%) and those who have received less or even only primary education in group 1 (62%).

**Figure 4:**
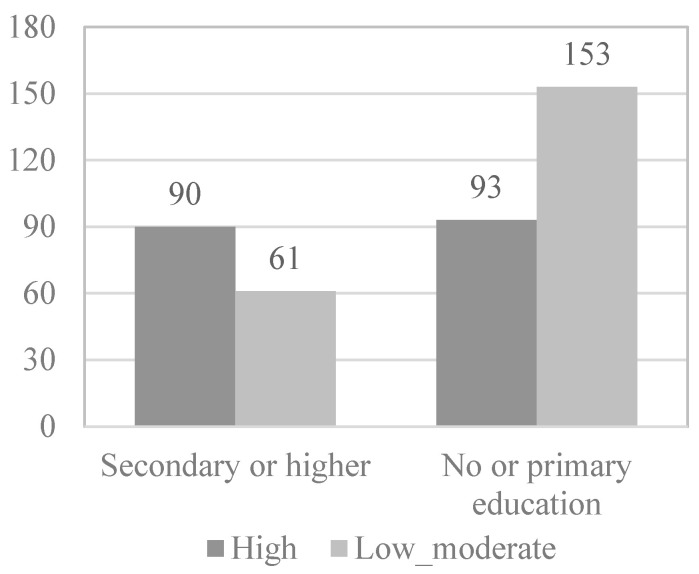
*Educational level* physical activity level.

Next, in the multivariate logistic regression model, the variables that obtained statistical significance in the bivariate analysis were introduced as variables: functional skills (*OR* = 2.527; *p* < 0.001), activity and leisure (*OR* = 4.110; *p* < 0.001), gender (*OR* = 1.905; *p* = 0.002), and educational level (*OR* = 2.427; *p* < 0.001) (Figure 1, Figure 2, Figure 3 and Figure 4).

Likewise, the interaction and/or confusion of the health variable with the functional abilities scale and with the activity and leisure scale was studied. The interaction health–activity and leisure was incorporated into the model, which showed significance (*OR* = 0.170; *p* = 0.001) because having low levels of health and of activity and leisure are predictive factors for having a low level of physical activity. The health variable was also incorporated into the model (*OR* = 1.378; *p* = 0.239). Furthermore, health is a confounding variable for functional abilities (*OR* = 3.006; *p* < 0.001). It was observed that the most explanatory variable of having a low/moderate level of physical activity is the activity and leisure variable (13.3 times higher for the elderly with a low level of activity and leisure) (Table 3).

In this model, the fit of the data is optimal: Hosmer and Lemeshow test > 0.05 and the likelihood ratio is significant (*χ*2 = 94.712; gl 6; *p* < 0.01). The effect size of the physical activity level model reached a Nagelkerke R2 value of 0.284, so it can explain 28% of the variability, being the model that explains the most variance. The model allows a correct estimation of 69.8% of the cases, with the sensitivity of the model being 70.6% and the specificity being 68.9%.

## 4. Discussion

Firstly, our results indicate that a low or moderate level of physical activity is related to a low functional skills score. Thus, this research confirms the relationship between the practice of low physical activity and the development or maintenance of the elderly person’s ability to perform activities of daily living, which, in turn, affects their quality of life. Other studies have shown similar results [27]. Therefore, an appropriate level of physical fitness is an important element for older people to maintain their autonomy [28]. However, these studies did not examine this topic considering the levels of physical activity practiced in the same way as the present study has done.

Secondly, it should be highlighted that a low-to-moderate level of physical activity practiced is related to low participation in activities and leisure time. These results show how necessary it is to increase leisure activities and free time in the elderly so that, indirectly, the levels of physical activity also increase. In addition, these findings indicate that performing a specific sporting activity or exercise in the elderly is a way to increase activities such as visiting friends, playing games, or running errands. In fact, the scientific literature points out the impact that physical activity has on greater social participation [29]. However, the present study provides one more specificity to the literature regarding a low-to-moderate level of physical activity and low participation in leisure activities, which leads us to conclude that leisure activities such as visiting friends, playing games, or running errands increase the practice of physical activity.

Thirdly, our results show the existence of a low-to-moderate level of physical activity practice in women, which makes it necessary to study the causes and motivations of this behaviour to improve their long-term health. However, and despite the lack of research in this regard, some studies mention that married women are more likely to perform more than 150 min of PA/week than single women, while there were no differences between married men, no matter their age [30]. However, various studies indicate that the differences in the effects that physical activity produces according to the gender of the participants are not clear [31], so it would be interesting to investigate this aspect further in relation to what this work has tried to do. In general, the scientific literature has focused more on the effects of physical activity on cancer in women [32] or cognitive function [33].

Fourth, this study shows that low-to-moderate levels of physical activity practiced among the participants are associated with primary studies, so the inference might be that knowledge of health can encourage the practice of physical activity in the elderly. Various investigations have also found a relationship between the practice of physical activity in older adults and certain social, cultural, and demographic matters [34]. In this regard, it has been found that a high level of education led 9896 older people to participate in activities associated with improving health and wellbeing [19]. In addition, 689 people older than 60 years with a good education adopted significantly stronger health behaviours [20]. These studies express the idea that a higher educational level can make individuals consider sport as an unavoidable part of health, and hence, its practice is associated with the search for better living conditions [35]. Despite what has been said, these results do not correspond to the differences in the level of physical activity practiced, as is the case in the current publication, which considers physically active older people and differentiates their high or low-to-moderate level of physical activity based on their educational level. This is because of the lack of resources that usually occurs among older adults who live in areas with a lower socioeconomic level, representing a population group that, although usually absent in research on physical activity, has been considered here. Indeed, the study of this sector is very necessary [36].

Fifth, this study has detected that the most explanatory variable of having a low/moderate level of physical activity is having a low level of activity and leisure. In this way, leisure activities in older people encourage the practice of physical activity; from which it follows that visiting friends, playing games, or running errands are an indirect way to increase the practice of physical activity, as has been exposed above. Once again, research in this regard is scarce; only one of them, carried out with 92 elderly people between 74 and 83 years old, showed that one of the factors influencing the practice of physical activity was social support, commitment, and networks [7].

Other matters that have been revealing in our study are the proximity or distance from a park, external conditions, and the structure of the neighbourhood, all of which can foster (or not) physical activity in 336 people over 60 years of age [31].

As for theoretical implications, it should be considered that the present results complement and update the scientific literature in the field, since they show that differences in functional ability are produced based on gender, educational level, and participation in leisure activities according to the level of physical activity practiced. These results suggest that it would be necessary to study why there is an increase in physical activity when there is a participation in leisure activities; in this way, they could be used as an indirect way to promote healthy living in older people.

Therefore, a practical implication of this research would be, precisely, the need to implement policies that mitigate or modulate the impact that variables such as gender or education have on the lesser practice of physical activity, through specific programs aimed at using activities of leisure such as visiting friends, playing games, or running errands as an indirect way to increase the practice of physical activity, as well as specific training to increase the level of awareness and adherence from close circles and government public entities, with the aim of promoting a greater practice of physical activity.

However, it should also be noted that one of the limitations of this study is the impossibility of generalizing its results, since the sample was only made up of active older people. A second hindrance is that the evaluation was carried out through a battery of self-administered questionnaires that, despite being recommended by the WHO to assess physical activity, do not contain objective measurement instruments such as, for example, a pedometer, along with the overestimation of responses on a self-administered questionnaire. Similarly, the cross-sectional nature of the study does not allow us to infer causal effects. Finally, it is important to emphasize that the scarcity of scientific studies on the elderly population that address the specificity of the different levels of physical activity has complicated the comparison between investigations. However, this limitation is, in turn, a strength since our study provides new information and complements the existing research with new results. Likewise, another strong point of the study has been the use of validated and adequate instruments. This study provides valuable knowledge in a population that is difficult to find (older people who practice physical activity) and reveals predictors of increased physical activity that could help to understand the elements that we should take into account from a scientific point of view and that favour greater physical activity. In fact, this study shows that low levels of education and being a woman hinder physical proactivity, with leisure activities or running errands being an indirect way to increase physical activity.

Taking these data into account, a future line of research could be to discover the characteristics of the environment and the sociodemographic variables that affect the practice of a low level of physical activity in vulnerable sectors of the population such as older women and, in general, older people with a low educational level.

## 5. Conclusions

As a general conclusion, it can be highlighted that the results of the present research show that gender, educational level, health, functional skills, and activity and leisure are variables that can predict the level of physical activity of older people who perform physical activity. These results reveal that there are components of the quality of life of older people that go beyond gender and education that can predict the level of physical activity in physically active older people. Thus, having a low level of education and being a woman makes it difficult to have proactive health behaviour. For this reason, it is important to practice physical activity, but it is also important to be aware of the level of activity practiced, where a high practice of physical activity favours a functional capacity of the elderly that can guarantee their autonomy and independence to carry out daily activities of life.

In addition, according to our results, older people who participate in leisure activities such as visiting friends, playing games, or running errands also tend to have a higher level of physical activity. Therefore, encouraging them to participate in activities and leisure can be an indirect way to increase their practice of physical activity. It is also necessary to study in depth the causes, problems, and motivations that make women practice less physical activity. All of this is to improve their quality of life. Finally, providing training and information for the elderly is necessary for the acquisition of healthy lifestyle habits and to achieve higher levels of physical practice.

## Figures and Tables

**Table 1:** Mean, standard deviations, and *t*-test of the scores on the scales of health, social integration, functional abilities, activity and leisure, and environmental quality according to the level of physical activity.

	Physical Activity Level					
*Scale*	High		Low-Moderate		*t*	*p*
	*n*	*M (SD)*	*n*	*M (SD)*		
Health	183	3.20 (0.41)	214	3.11 (0.54)	1.915	0.056
Social Integration	183	3.31 (0.40)	214	3.35 (0.40)	−1.002	0.317
Functional skills	183	3.79 (0.34)	214	3.60 (0.47)	4.819	0.000 *
Activity and leisure	183	2.99 (0.43)	214	2.64 (0.39)	8.459	0.000 *
Environment quality	183	2.97 (0.09)	214	2.96 (0.09)	0.510	0.610

Note * *p* < 0.05. *n* = number of participants; *M* = mean; *SD* = standard deviation; *t* = t-student; *p* = *p*-value.

**Table 2:** Frequencies of gender, level of studies, and use of socio-health services according to the level of physical activity of the participants.

	Physical Activity Level					
	High		Low-Moderate		Total	
	*n*	*%*	*n*	*%*	*n*	*%*
*Gender*						
Male	79	43.2	61	28.5	140	35.3
Female	104	56.8	153	71.5	257	64.7
*Education level*						
Less than primary studies	39	21.3	104	48.6	143	36.0
Primary studies	54	29.5	49	22.9	103	25.9
VET and high school	43	23.5	29	13.6	72	18.1
Middle/university degree	47	25.7	32	15.0	79	19.9
*Use of social and health services*						
Never	12	6.6	12	5.6	24	6.0
Occasionally	107	58.5	119	55.6	226	56.9
Frequently	64	35.0	83	38.8	147	37.0

Note *p* < 0.05; *n* = number of participants; % = percentage.

**Table 3:** Logistic regression model of physical activity level.

	B	S.E.	Wald	*p*	Exp(B)	IC 95 for Exp(B)
Gender	0.751	0.243	9.553	0.002 *	2.120	1.316–3.413
Education level	0.619	0.246	6.314	0.012 *	1.856	1.146–3.007
Health	−0.316	3.09	1.050	0.306	0.729	0.398–1.335
Functional skills	0.992	0.260	14.568	0.000 *	2.695	1.620–4.485
Activity and leisure	2.586	0.454	32.380	0.000 *	13.274	5.447–32.344
Health–Activity and Leisure	−1.818	0.548	11.001	0.001 *	0.162	0.055–0.475
Constant	−1.547	0.277	31.188	0.000 *	0.213	

Note * *p* < 0.05; B = Coefficient b; S.E. = standard error; Wald = the Wald test; *p* = *p*-value; Exp(B) = Odds Ratio; IC95 for Exp(B) = 95% Confidence interval.

## Data Availability

The data is in the custody of the PI.
